# Production of hemolysin BL by *Bacillus cereus* group isolates of dairy origin is associated with whole-genome phylogenetic clade

**DOI:** 10.1186/s12864-016-2883-z

**Published:** 2016-08-09

**Authors:** Jasna Kovac, Rachel A. Miller, Laura M. Carroll, David J. Kent, Jiahui Jian, Sarah M. Beno, Martin Wiedmann

**Affiliations:** Department of Food Science, Cornell University, Ithaca, NY 14853 USA

**Keywords:** *Bacillus cereus* group, WGS, Virulence genes, Dairy, Toxin production, Hemolysin BL

## Abstract

**Background:**

*Bacillus cereus* group isolates that produce diarrheal or emetic toxins are frequently isolated from raw milk and, in spore form, can survive pasteurization. Several species within the *B. cereus* group are closely related and cannot be reliably differentiated by established taxonomical criteria. While *B. cereus* is traditionally recognized as the principal causative agent of foodborne disease in this group, there is a need to better understand the distribution and expression of different toxin and virulence genes among *B. cereus* group food isolates to facilitate reliable characterization that allows for assessment of the likelihood of a given isolate to cause a foodborne disease.

**Results:**

We performed whole genome sequencing of 22 *B. cereus* group dairy isolates, which represented considerable genetic diversity not covered by other isolates characterized to date. Maximum likelihood analysis of these genomes along with 47 reference genomes representing eight validly published species revealed nine phylogenetic clades. Three of these clades were represented by a single species (*B. toyonensis* –clade V*, B. weihenstephanensis* – clade VI*, B. cytotoxicus* - VII), one by two dairy-associated isolates (clade II; representing a putative new species), one by two species (*B. mycoides, B. pseudomycoides* – clade I) and four by three species (*B. cereus, B. thuringiensis, B. anthracis* – clades III-a, b, c and IV). Homologues of genes encoding a principal diarrheal enterotoxin (hemolysin BL) were distributed across all, except the *B. cytotoxicus* clade. Using a lateral flow immunoassay, hemolysin BL was detected in 13 out of 18 isolates that carried *hblACD* genes. Isolates from clade III-c (which included *B. cereus* and *B. thuringiensis*) consistently did not carry *hblACD* and did not produce hemolysin BL. Isolates from clade IV (*B. cereus, B. thuringiensis*) consistently carried *hblACD* and produced hemolysin BL. Compared to others, clade IV was significantly (*p* = 0.0001) more likely to produce this toxin. Isolates from clade VI (*B. weihenstephanensis*) carried *hblACD* homologues, but did not produce hemolysin BL, possibly due to amino acid substitutions in different toxin-encoding genes.

**Conclusions:**

Our results demonstrate that production of diarrheal enterotoxin hemolysin BL is neither inclusive nor exclusive to *B. cereus* sensu stricto, and that phylogenetic classification of isolates may be better than taxonomic identification for assessment of *B. cereus* group isolates risk for causing a diarrheal foodborne disease.

**Electronic supplementary material:**

The online version of this article (doi:10.1186/s12864-016-2883-z) contains supplementary material, which is available to authorized users.

## Background

*Bacillus cereus* was identified as a causative agent in 19 % of foodborne outbreaks that were reported in the United States from 1998 to 2008 [1]. Although the majority of these outbreaks were traced back to rice (50 %) and meat (24 %) [1], *B. cereus* group isolates have also been frequently isolated from milk e.g., [2, 3]. *B. cereus* spores can survive high temperature exposure, therefore insufficient cooling or storage of food at temperatures below 60 °C can support their growth in food following thermal treatment. Depending on the strain, growth to high levels may result in food spoilage and present a risk for foodborne illness after ingestion [4, 5].

The *B. cereus* group consists of eight pathogenic and non-pathogenic bacterial species – *B. cereus*, *B. anthracis, B. thuringiensis, B. cytotoxicus, B. weihenstephanensis, B. mycoides, B. pseudomycoides,* and *B. toyonensis* [6, 7]. This group of bacterial species is also referred to as *B. cereus* sensu lato with the specific species *B. cereus* being referred to as *B. cereus* sensu stricto. Members of the *B. cereus* group are closely related and cannot always be differentiated based on phenotypic and biochemical characteristics as detailed in the Bergey’s Manual of Determinative Bacteriology [8]. Nevertheless, some specific phenotypic characteristics have traditionally been used to differentiate key species within the *B. cereus* group. For example, a combination of capsule production, non-motility and inability to cause hemolysis is specific for *B. anthracis*, the production of crystal proteins is specific for *B. thuringiensis*, and rhizoid colony morphology is specific for *B. mycoides* and *B. pseudomycoides* [8]. In addition, the ability to grow at 7 °C but not at 43 °C is typically considered to be specific for *B. weihenstephanensis* [8]. Even with molecular methods, species classification *of B.* cereus group isolates can be challenging. For example, the human pathogens *B. cereus* and *B. anthracis*, and the insect pathogen *B. thuringiensis* cannot be reliably differentiated with most molecular typing methods, including DNA sequence analysis of 16S rDNA, *rpoB* and MLST loci [3, 9, 10]. Consistent with these findings, studies employing whole genome sequencing have confirmed high genomic similarity of *B. cereus, B. thuringiensis* and *B. anthracis*, which explains the difficulties in speciation of *B. cereus* group isolates [11–14].

Due to the highly similar genomic backbone of *B. cereus* group isolates, different studies have explored whether characterization of virulence gene presence/absence patterns may provide a better predictor of a strain’s ability to cause anthrax or gastrointestinal disease, compared to traditional phenotypic or molecular taxonomic classification. These methods typically determine the presence/absence of *B. cereus* group virulence genes encoded on plasmids or within the chromosome, which were previously linked to virulence in humans and/or animals. Among plasmid-encoded virulence genes are those encoding the anthrax toxin (*cya, lef* and *pag*) and the poly-γ-D-glutamate capsule biosynthesis genes (*capABCDE*) [12, 15]. These virulence determinants are encoded on pXO1, pXO2 or similar plasmids, which have been identified among *B. anthracis* isolates, as well as *B. cereus* isolates able to cause anthrax or anthrax-like disease in humans and animals [15–20]. The *cesABCD* operon represents another set of virulence genes encoded on plasmids (e.g., pCERE01, pBCE4810). These genes encode cereulide synthetase necessary for non-ribosomal biosynthesis of the emetic toxin cereulide, which causes a food-borne intoxication in humans [21, 22]. Lastly, strains carrying plasmid-encoded crystal protein genes (*cry*) are pathogenic to insects, but crystal proteins do not affect humans [23, 24].

In contrast, genes encoding the virulence determinants implicated in the diarrheal type of the foodborne disease are typically chromosomally encoded. Examples include hemolysin BL (encoded by *hblABCD*), nonhemolytic enterotoxin (*nheABC*), cytotoxin K (*cytK*) and cereolysin (*cerAB*) [24, 25]. Genes encoding diarrheal toxins are distributed across different *B. cereus* group species, including *B. cereus*, *B. thuringiensis*, *B. weihenstephanensis, B. mycoides* and *B. pseudomycoides* [26–29]. Expression of these toxins is governed by complex regulation pathways, which are not well understood [29–32]. In addition to these principal virulence genes, multiple other genes exist, which may indirectly contribute to virulence, such as *hlyII, entFM* and *entABC*, which encode hemolysin II, enterotoxin FM, and a putative enterotoxin, respectively [25].

Overall, classification into currently defined species within the *B. cereus* group does not provide for reliable identification of isolates that are or are not likely to cause human foodborne disease. For example, production of diarrheal toxins in the *B. cereus* group is not species - specific. While some attempts have been made to use single locus (*panC*) sequence data to develop alternative classification approaches that allow for more accurate assessment of pathogenicity and virulence of *B. cereus* group isolates [33, 34], these approaches still require significant refinement and further data. In particular, the accuracy of classification into seven phylogenetic groups, as determined based on *panC* sequences needs to be confirmed using whole genome sequences. The diversity of *B. cereus* group isolates evaluated to-date by WGS has included only a limited number of food isolates and, to our knowledge, few genome sequences for dairy isolates are publicly available. However, sporeforming pathogens and spoilage organisms are becoming increasingly important in dairy products as reduced post-processing contamination with rapidly growing Gram-negative bacteria provide an opportunity for growth of *B. cereus* group isolates that would not compete well in the presence of many Gram-negative bacteria introduced by post-processing contamination. Hence, we have carried out a comparative genomic analysis of 22 dairy-associated *B. cereus* group isolates and 47 *B. cereus* group isolates with publicly available genome sequences to investigate their (i) phylogenetic clustering based on core genome single nucleotide polymorphisms (SNPs), (ii) clustering based on the virulence gene presence/absence, and (iii) distribution of hemolysin BL and nonhemolytic toxin production among isolates in different phylogenetic clades.

## Results and discussion

### Identification of multiple novel MLST allelic and sequence types reflects the diversity of dairy-associated *B. cereus* group isolates

Whole genome sequencing was performed for 22 diverse dairy-associated isolates (Table 1) that had been identified as members of *B. cereus* group based on *rpoB* sequence data. The median whole genome sequence (WGS) coverage for the 22 isolates was 57-fold (ranging from 33 to 78-fold), the median number of contigs >1 kb was 128 (ranging from 63 to 846; only one sequence, FSL W8-0767 was on the higher end), and the median draft genome size was 5.75 Mbp.

**Table 1:** Characteristics and sequence accession numbers for dairy-associated *B. cereus* group isolates sequenced in this study

Isolate	Source	BioSample ID	SRA Run	GenBank accession number	PubMLST ID	*rpoB* allelic type (AT)	MLST ST^a^	Novel MLST AT or ST^b^
FSL H7-0926	Pasteurized 2 % milk	SAMN03800014	SRR2541537	LOBD00000000	1774	90	667	
FSL H8-0482	Soil from areas adjacent to barns	SAMN03800015	SRR2541601	LOAZ00000000	1764	129	223	
FSL H8-0488	Water from hose in milking area	SAMN03800016	SRR2541602	LOBA00000000	1765	129	111	
FSL K6-0040	Raw milk	SAMN03800017	SRR2541603	LOBE00000000	1775	362	1101	*ilv* 245, ST-1101
FSL K6-0043	Raw milk	SAMN03800018	SRR2541604	LOMW00000000	1763	363	1087	*pta* 235, ST-1087
FSL K6-0067	Raw milk	SAMN03800019	SRR2541605	LOMN00000000	1762	365	1086	*gmk* 138, *pur* 218, ST-1086
FSL K6-0069	Raw milk	SAMN03800020	SRR2541606	LOBB00000000	1756	194	1080	*glp* 224, *gmk* 135, *ilv* 237, *pta* 233, ST-1080
FSL K6-0073	Raw milk	SAMN03800021	SRR2541607	LOMO00000000	1773	366	33	
FSL K6-0267	Raw milk	SAMN03800022	SRR2541613	LOMP00000000	1755	90	1090	ST-1090
FSL M8-0117	Raw milk	SAMN03800023	SRR2541639	LONG00000000	1761	308	1085	*gmk* 137, *pyc* 186, ST-1085
FSL W8-0003	Ricotta/mozzarella whey	SAMN03800024	SRR2541640	LOMQ00000000	1760	125	1084	*ilv* 239, ST-1084
FSL W8-0050	Condensed product from evaporator	SAMN03800025	SRR2541641	LOMR00000000	1772	380	32	
FSL W8-0169	Raw milk	SAMN03800026	SRR2541651	LOBC00000000	1757	61	1081	*gmk* 136, *pta* 234, ST-1081
FSL W8-0268	Evaporator - liquid	SAMN03800027	SRR2541662	LOMS00000000	1759	92	1083	*tpi* 185, ST-1083
FSL W8-0275	Liquid milk whey permeate	SAMN03800028	SRR2541668	LOMT00000000	1771	463	1050	
FSL W8-0483	Liquid raw milk from silo	SAMN03800029	SRR2541674	LOMU00000000	1758	120	1082	*ilv* 238, ST-1082
FSL W8-0520	Processed liquid milk	SAMN03800030	SRR2541680	LONH00000000	1770	481	1032	
FSL W8-0523	Liquid from blended whey silo	SAMN03800031	SRR2541686	LOMV00000000	1769	481	1032	
FSL W8-0640	Processed skim milk	SAMN03800032	SRR2541688	LOQA00000000	1754	154	1089	ST-1089
FSL W8-0824	Finished dairy product	SAMN03800033	SRR2541693	LOQB00000000	1767	92	24	
FSL W8-0932	Processed skim milk	SAMN03800034	SRR2541715	LOQC00000000	1766	120	365	
FSL W8-0767	Milk powder	SAMN03800035	SRR2541718	LOQD00000000	1768	154	787	

^a^ST sequence type, MLST STs were assigned to be consistent with the *B. cereus* PubMLST database [35]

^b^AT allelic type; this column indicates new allelic types for MLST genes (e.g., *ilv* 245; gene names can be found on PubMLST database website [35]) and newly assigned STs (e.g., ST-1087)

WGS data were initially used to extract sequence data for seven genes that are used in the *B. cereus* PubMLST typing scheme [35]. MLST data differentiated the 22 isolates into 21 different MLST sequence types (STs), providing increased discriminatory power over *rpoB* sequence typing that identified 16 different *rpoB* allelic types (ATs). Eleven of these MLST types represented novel STs in the PubMLST database (Table 1) [35]. Two of them were a novel combination of existing ATs and nine of them carried one to four novel allelic types, which were deposited in the PubMLST database. These data show that the isolates selected represent considerable *B. cereus* group strain diversity that has not been observed among isolates characterized by MLST or WGS to date. These data also demonstrate the importance of investigating populations from diverse sources when characterizing the genomic diversity of different bacterial groups. These initial analyses further justify our investigation of the genomic diversity among dairy-associated *B. cereus* group isolates, particularly since previous studies focused on clinical sources and food sources most commonly linked to human disease outbreaks (e.g., rice, vegetables) (e.g., [36, 37]).

### WGS-based phylogeny reveals clades consistent with *rpoB* and MLST clustering of isolates

**Fig. 1:**
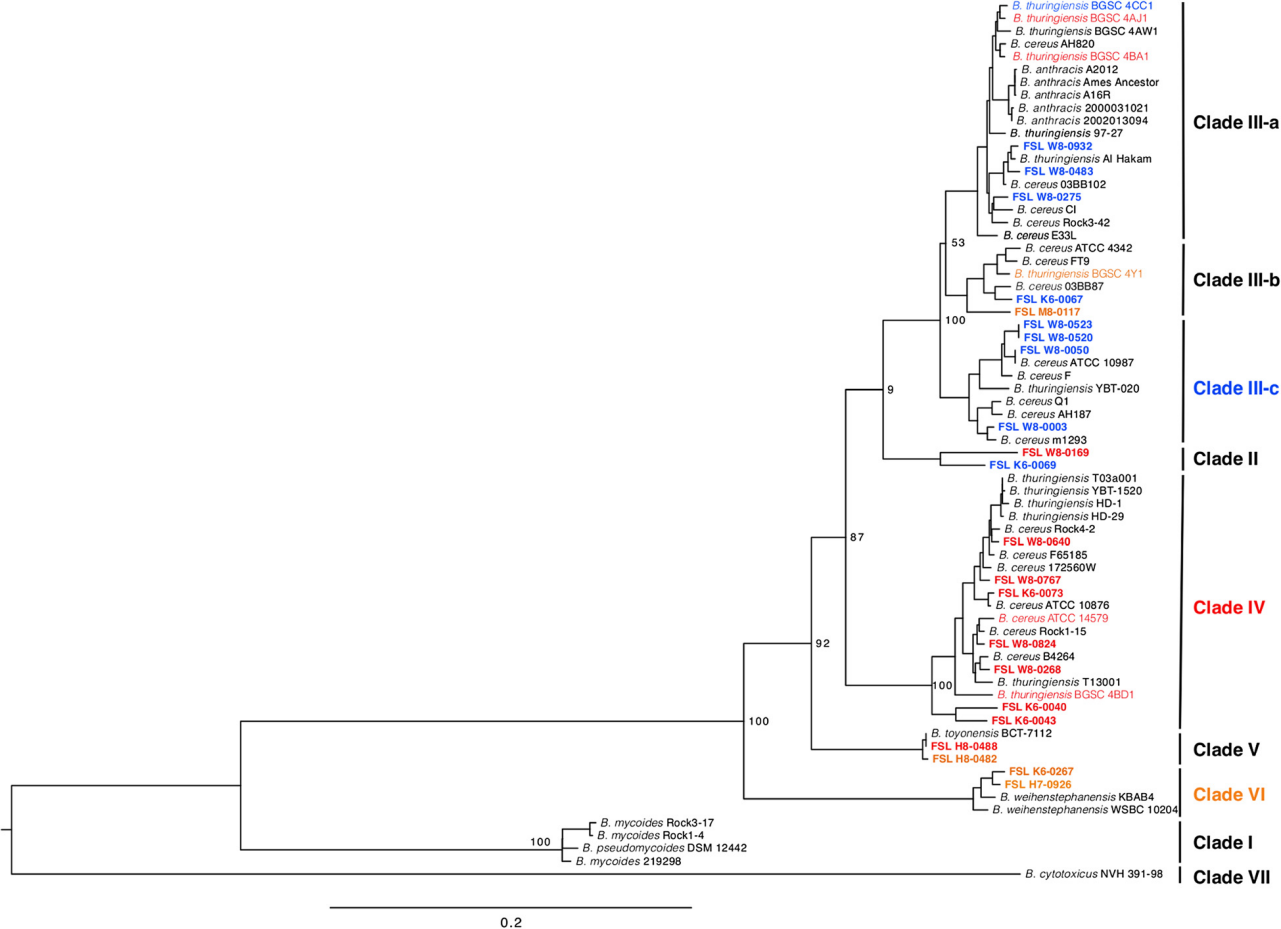
Core genome phylogeny of 69 *B. cereus* group isolates. Maximum likelihood tree was constructed using core genome SNPs identified with kSNP for 22 dairy-associated isolates and 47 reference isolates. Phylogeny was inferred using RaxML under general time-reversible model with gamma distributed substitution sites, and 1000 bootstrap repetitions. Bar represents 0.2 substitutions per site. WGS revealed nine *B. cereus* group phylogenetic clades. These clades were named according to previously proposed phylogenetic groups based on *panC* sequence types [33]. Where *panC* phylogenetic groups could be separated into different WGS clades this was clarified by the use of alphabetical subdesignation (e.g., *panC* group III was resolved into WGS clades III-a, III-b, and III-c). Isolates in red carried *hblACD* genes and produced hemolysin BL, isolates in orange carried *hblACD* genes, but did not produce hemolysin BL, and isolates in blue did not carry *hblACD* genes nor did they produce hemolysin BL. Dairy-associated isolates are in bold

**Fig. 2:**
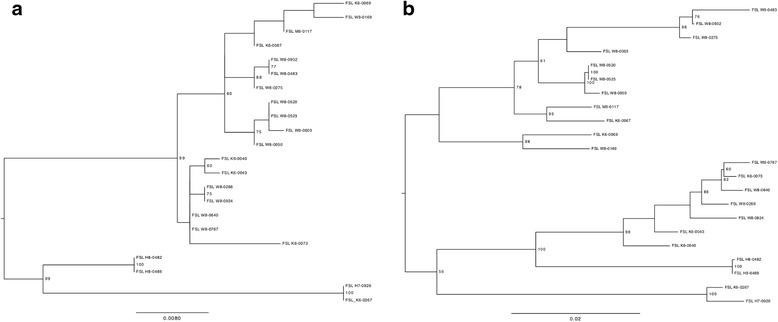
Maximum likelihood phylogenetic tree constructed based on (**a**) *rpoB* and (**b**) 7 MLST loci. Maximum likelihood tree constructed in RaxML under general time-reversible model with gamma distributed and invariant substitution sites, and 1000 bootstrap repetitions, based on (**a**) *rpoB* and (**b**) 7 MLST loci sequences for 22 dairy-associated isolates. Bar represents (**a**) 0.02 and (**b**) 0.0080 substitutions per site. Only bootstrap values ≥ 60 are shown on the trees

Genome sequence data for the 22 dairy-associated isolates and 47 reference genomes (obtained from NCBI, RefSeq; see Additional file 1: Table S1) of *B. cereus* group isolates were used to build a core genome kSNP tree (Fig. 1), which was based on 2362 core genome SNPs. The resulting phylogeny was compared to clustering of the 22 dairy-associated isolates that was observed in separate phylogenies constructed with either *rpoB* or the 7 MLST genes sequence data. WGS revealed nine *B. cereus* group clades. These clades were named according to previously proposed phylogenetic groups based on *panC* sequences [33]. Where *panC* phylogenetic groups could be separated into different WGS clades this was clarified by use of alphabetical subdesignation (e.g., *panC* group III was resolved into WGS clades III-a, III-b, and III-c). Seven of WGS clades (i.e., clades II to VI) included between two and seven dairy-associated isolates sequenced here. While the 22 dairy-associated isolates also clustered in seven clades in the MLST tree, these isolates represented only 6 phylogenetic clades in the *rpoB* tree. Specifically, one of the clades in the *rpoB* tree was resolved into two clades in MLST tree, including one clade with isolates FSL M8-0117 and FSL K6-0067, and one with FSL K6-0069 and FSL W8-0169 (Fig. 2). Clustering of dairy-associated isolates in the *rpoB* and WGS trees was very similar, but not absolutely congruent, as the *rpoB* tree did not resolve clades II and III-b (Fig. 2). The WGS phylogeny was congruent with MLST phylogeny, and with the seven phylogenetic groups determined based on *panC* sequence similarity (see Fig. 1), which were proposed by Guinebretière et al. [33, 34]. Overall, these data suggest that both *rpoB* and *panC* single-locus-based characterization allows for classification that is expected to be largely congruent with WGS clades.

### Genomic sequence data confirm that the *B. cereus* group includes both monophyletic species, as well as currently designated species that are not consistent with phylogeny

The WGS-based phylogeny constructed here includes whole genome sequence (WGS) data for 47 reference genomes of *B. cereus* group isolates that had been previously sequenced and identified at the species level by others (i.e., *B. cereus, B. anthracis, B. cytotoxicus, B. thuringiensis, B. weihenstephanensis, B. mycoides, B. pseudomycoides, B. toyonensis*). Metadata for these reference isolates are available in Additional file 1: Table S1. Among nine clades defined here, all but clade II (representing a putative new species) included at least one reference strain. Three of the WGS clades (V, VI and VII) included reference strains of only a single species, including *B. toyonensis* (clade V), *B. weihenstephanensis* (clade VI) and *B. cytotoxicus* (clade VII). While only one reference strain genome was available for each *B. toyonensis* and *B. cytotoxicus,* both *B. weihenstephanensis* reference genomes analyzed clustered into clade VI. Clades V and VI each, also included dairy-associated isolates. All three of these clades are supported by 100 % bootstrap values. These data suggest that *B. toyonensis*, *B. weihenstephanensis* and *B. cytotoxicus* represent monophyletic species. Consistently with our findings, *B. cytotoxicus* has been previously classified in a separate phylogenetic group [33].

Clade I included three genomes previously identified as *B. mycoides*, as well as the *B. pseudomycoides* type strain genome. It has previously been shown that *B. mycoides* and *B. pseudomycoides* isolates (identified based on rhizoid colony morphology) cluster in two MLST clades – one including the *B. pseudomycoides* type strain, and the other including *B. weihenstephanensis* and *B. mycoides* type strains [38]. Clustering of previously identified (reference) *B. mycoides* genomes with genome of *B. pseudomycoides* type strain in our study suggests that these *B. mycoides* isolates were originally misclassified, and should be re-classified as *B. pseudomycoides*. Rhizoid colony morphology is typical for both *B. mycoides* and *B. pseudomycoides* and is therefore not an appropriate differentiating characteristic. Classification based on *panC* sequence analysis is more reliable, as *B. pseudomycoides* cluster in *panC* group I, and *B. mycoides* in *panC* group VI [33].

Each of the remaining four clades (III-a, III-b, III-c and IV) included both *B. cereus* and *B. thuringiensis* reference genomes. In addition to *B. cereus* and *B. thuringiensis*, clade III-a also included all 5 *B. anthracis* reference genomes from our isolate set. These results are consistent with MLST studies that have also shown (i) that *B. cereus* and *B. thuringiensis* cluster together (e.g., [9]) and (ii) that *B. anthracis* isolates cluster with *B. cereus* (e.g., [39]). Overall, our data confirmed the polyphyletic nature of the species *B. cereus* and *B. thuringiensis* by demonstrating their clustering in multiple “multi-species” clades, similar to what was previously reported based on the WGS analyses [11–14]. This shows that WGS phylogeny does not support the current taxonomical classification of *B. cereus, B. thuringiensis* and *B. anthracis*, and suggests that SNP-based phylogenetic clustering, even at the core genome level, does not allow for identification of these species the way they are defined now. Similar to our findings, previous MLST studies have shown that clustering of *B. cereus* group isolates does not yield clades that are consistent with species designation [9, 38, 39]. There is thus increasing and consistent evidence that supports the need for a re-alignment of the *B. cereus* group taxonomy to be consistent with the WGS phylogeny. The data generated here can form the basis for future development of SNP-based assays that will allow for rapid identification of major *B. cereus* group clades and subtypes.

### Insecticidal crystal protein genes were not detected in all isolates previously identified as *B. thuringiensis*, and were detected in isolates representing other species

In order to further characterize the genomes analyzed here, we determined the presence/absence of 36 virulence genes (Additional file 2: Table S2 and Additional file 3: Table S3) in the whole genome sequences of the 69 *B. cereus* group genomes using protein BLAST. The presence of 8 of these 36 virulence genes was confirmed by performing PCR on 28 *B. cereus* group isolates that were physically available to us (Table 2). While standard protein BLAST approach was generally successful in identifying the genomic presence of virulence genes (see below for details), *cry*, which encodes the insecticidal crystal protein typically linked to B*. thuringiensis*, showed substantially higher amino acid variability than other virulence-associated genes [40]. As this increases the chances of false negative results using protein BLAST, we initially searched for *cry* using an Endotoxin_M hidden Markov model (HMM) extracted from Pfam database (PF00555). This HMM search identified *cry* homologues in just 4 out of 14 *B. thuringiensis* genomes. To improve the sensitivity of the HMM search, we thus constructed a custom Hidden Markov model based on ten *cry* variants representing different 1° domains available in the *B. thuringiensis* nomenclature database [41]. This new model identified *cry* genes in 13 isolates (Additional file 3: Table S3), including the same 4 isolates that were positive for *cry* with the Enterotoxin_M HMM search. These 13 isolates represented *B. thuringiensis* (10/14 reference genomes), *B. weihenstephanensis* (1 reference genome)*,* and two dairy-associated *B. cereus* group isolates. All genomes were also annotated with RAST [42], which identified *cry* genes in four *B. thuringiensis* isolates, three dairy-associated isolates and a single *B. weihenstephanensis* isolate. Overall, *cry* genes were detected in 16 isolates by at least one of the approaches used (Table 3, Additional file 3: Table S3). The challenges with detection of *cry* genes are consistent with previous reports that these genes are highly diverse (e.g., [23]) and demonstrate a need for further development of improved tools for detection of *cry* genes based on WGS data. Interestingly, isolates identified as carrying *cry* genes were distributed across clades III-a (*n* = 3), III-b (*n* = 2), III-c (*n* = 1), IV (*n* = 8), V (*n* = 1) and VI (*n* = 1), indicating that *cry* homologs are widely distributed across different clades of *B. cereus* group isolates.

**Table 2:** Distribution of key virulence genes among *B. cereus* group species and WGS clades

	% Isolates that carry virulence genes^a^																	
Species	*cry*	*cya + lef + pag*	*capA*	*capB*	*capC*	*capD*	*capE*	*cesA*	*cesB*	*cesC*	*cesD*	*cytK*	*entFM*	*hblA*	*hblB*	*hblC*	*hblD*	*nheABC*
*B. anthracis* (*n* = 5)		60	80	80	80	80	80						100					100
*B. cereus* (*n* = 20)		15	15	5	15	15	10	5	5	5	5	70	95	50	45	45	50	100
*B. cytotoxicus* (*n* = 1)												100	100					100
*B. mycoides* (*n* = 3)												33	100	100	67	100	100	100
*B. pseudomycoides* (*n* = 1)													100	100	100			100
*B. thuringiensis* (*n* = 14)	71		7		7	7						79	100	86	86	86	86	100
*B. toyonensis* (*n* = 1)							100			100			100	100	100	100	100	100
*B. weihenstephanensis* (*n* = 2)	50												100	100	50	100	100	100
Dairy-associated isolates (*n* = 22)	9											68	100	59	50	59	59	100
WGS clade																		
Clade I (*n* = 4)													100	100	75	75	75	100
Clade II (*n* = 2)												100	100	50	50	50	50	100
Clade III-a (*n* = 19)	16	26	37	26	37	37	32					47	100	26	26	26	26	100
Clade III-b (*n* = 6)	33	17										83	83	67	67	67	67	100
Clade III-c (*n* = 10)	10							10	10	10	10	50	100	10	10	10	10	100
Clade IV (*n* = 20)	40		5		5	5						95	100	100	95	95	100	100
Clade V (*n* = 3)	33						33			33			100	100	100	100	100	100
Clade VI (*n* = 4)	25												100	100	25	100	100	100
Clade VII (*n* = 1)												100	100					100

^a^ Virulence genes were identified in whole genome sequences using BLAST

**Table 3:** Toxin gene presence, hemolysin BL (HBL) and nonhemolytic enterotoxin (NHE) production and hemolytic potential

Isolate	Virulence genes presence [PCR]^a^								Virulence genes presence [WGS]^a, b^								Production of toxin^a, c^		Hemolysis^d^
	*hblA*	*hlbC*	*hblD*	*nheA*	*nheB*	*nheC*	*ctyK*	*entFM*	*hblA*	*hblC*	*hblD*	*nheA*	*nheB*	*nheC*	*ctyK*	*entFM*	HBL	NHE	
FSL H7-0926	1	1	1	1	1	1	1	1	1	1	1	1	1	1	0	1	0	1	B
FSL H8-0482	1	1	1	1	1	1	0	1	1	1	1	1	1	1	0	1	0	1	B
FSL H8-0488	1	1	1	1	0	1	0	1	1	1	1	1	1	1	0	1	1	1	B
FSL K6-0040	1	1	1	1	1	1	1	1	1	1	1	1	1	1	1	1	1	1	B
FSL K6-0043	1	1	1	1	1	1	1	1	1	1	1	1	1	1	1	1	1	1	B
FSL K6-0067	0	0	0	1	1	1	1	1	0	0	0	1	1	1	1	1	0	1	-
FSL K6-0069	0	0	0	1	1	1	1	1	0	0	0	1	1	1	1	1	0	1	B
FSL K6-0073	1	1	1	1	1	1	1	1	1	1	1	1	1	1	1	1	1	1	A
FSL K6-0267	1	1	1	1	1	1	1	1	1	1	1	1	1	1	0	1	0	1	A
FSL M8-0117	1	1	1	1	1	1	1	1	1	1	1	1	1	1	1	1	0	1	A
FSL W8-0003	0	0	0	1	1	1	0	1	0	0	0	1	1	1	0	1	0	1	-
FSL W8-0050	0	0	0	1	1	1	1	1	0	0	0	1	1	1	1	1	0	1	B
FSL W8-0169	1	1	1	1	1	1	1	1	1	1	1	1	1	1	1	1	1	1	B
FSL W8-0268	1	1	1	1	1	1	1	1	1	1	1	1	1	1	1	1	1	1	B
FSL W8-0275	0	0	0	1	1	1	1	1	0	0	0	1	1	1	1	1	0	1	B
FSL W8-0483	0	0	0	1	1	1	0	0	0	0	0	1	1	1	0	1	0	1	B
FSL W8-0520	0	0	0	1	1	1	1	1	0	0	0	1	1	1	1	1	0	1	B
FSL W8-0523	0	0	0	1	1	1	1	1	0	0	0	1	1	1	1	1	0	1	B
FSL W8-0640	1	1	1	1	1	1	1	1	1	1	1	1	1	1	1	1	1	1	B
FSL W8-0824	1	1	1	1	1	1	1	1	1	1	1	1	1	1	1	1	1	1	B
FSL W8-0932	0	0	0	1	1	1	0	1	0	0	0	1	1	1	0	1	0	1	A
FSL W8-0767	1	1	1	1	1	1	1	1	1	1	1	1	1	1	1	1	1	1	A
*B. thuringiensis* BGSC 4Y1	1	1	1	1	1	1	1	1	1	1	1	1	1	1	1	1	0	1	B
*B. thuringiensis* BGSC 4CC1	0	0	0	1	1	1	1	1	0	0	0	1	1	1	1	1	0	1	B
*B. thuringiensis* BGSC 4AJ1	1	1	1	1	0	1	1	1	1	1	1	1	1	1	1	1	1	1	B
*B. thuringiensis* BGSC 4BD1	1	1	1	1	1	1	0	1	1	1	1	1	1	1	0	1	1	1	B
*B. thuringiensis* BGSC 4BA1	1	1	1	1	1	1	1	1	1	1	1	1	1	1	1	1	1	1	B
*B. cereus* ATCC 14579	1	1	1	1	1	1	1	1	1	1	1	1	1	1	1	1	1	1	B

^a^ 0 indicates absence of a gene; 1 indicates presence of a gene

^b^ WGS whole genome sequence

^c^ HBL hemolysin BL, NHE nonhemolytic enterotoxin

^d^ “-“ indicates non-hemolytic; “A” indicates α hemolysis, “B” indicates β hemolysis

### Anthrax toxin genes were found in isolates previously identified as *B. anthracis,* as well as *B. cereus*

Protein BLAST identified the anthrax toxin genes *cya, lef* and *pagA* in (i) 3 of the 5 *B. anthracis* reference genomes (strains 2000031021 and 2002013094 did not have these genes) and, (ii) 3 out of 20 *B. cereus* reference genomes (03BB102, CI and 03BB87). Strain CI was previously designated as *B. cereus* biovar anthracis because of the presence of anthrax virulence genes [15]. The complete *capABCDE* operon, which encodes the components for poly-γ-D-glutamate capsule biosynthesis, was found in (i) 4 of the 5 *B. anthracis* reference genomes (strain A16R did not have these genes) and, (ii) in 1 of the 20 *B. cereus* genomes (*B. cereus* biovar anthracis strain Cl). Hence, *B. cereus* strain CI carried a full complement of *B. anthracis* virulence genes. In addition, an incomplete *capABCDE* operon, characterized by the absence of *capB,* was detected in *B. cereus* strains 03BB102 and F65185, as well as in *B. thuringiensis* BGSC 4AJ1. *B. toyonensis* BCT-7112 carried only *capE* (Additional file 3: Table S3). These findings are consistent with previous reports of *B. cereus* strains isolated from patients with anthrax-like symptoms [16–18]. Interestingly, two *B. cereus* strains that carried anthrax genes (isolates 03BB102, CI) were found in clade III-a, which contains all *B. anthracis* reference genomes, while *B. cereus* 03BB87 was found in sister clade III-b.

### Diarrheal toxin genes were identified across multiple *B. cereus* group species

A protein BLAST was used to identify genes previously reported as encoding (i) metabolic pathways for production of cereulide linked to emetic illness and (ii) toxins linked to diarrheal illness. The cereulide synthetase gene cluster (*cesABCD*) was found in only one isolate, *B. cereus* AH187 from WGS clade III-c. Genes encoding toxins linked to diarrheal illness were found in a larger number of genomes. These genes included (i) *hblABCD* operon, (ii) *nheABC* operon and (iii) *cytK*.

(i) Genes encoding hemolysin BL (*hblABCD*)

Two different *hbl* operons have been identified previously [43]. One operon (*hbl*-I) consists of two genes encoding hemolysin BL binding component (*hblA –* 1125 nt, and *hblB –* 1398 nt), one gene encoding lytic component L2 (*hblC*), and one gene encoding lytic component L1 (*hblD*). The second operon (*hbl*-II) consists of a single gene encoding a binding component (*hblA*) and the two genes encoding lytic components (*hblC* and *hblD*) of hemolysin BL. Both of these operons have been previously reported in *B. cereus* strain ATCC 10876 [43]. We have identified *hblACD* genes encoding hemolysin BL in 10/20 *B. cereus* isolates (including one that did not carry *hblC*), 12/14 *B. thuringiensis* isolates, all 3 *B. mycoides* isolates, both *B. weihenstephanensis* isolates, the only *B. toyonensis* isolate and 13 dairy-associated isolates (Table 2). The *B. pseudomycoides* isolate carried only a *hblA*. Thirty-seven out of 42 isolates that carried a gene encoding the hemolysin BL binding component *hblA*, also carried a longer variant of binding component-encoding gene, *hblB*. Four Hbl-encoding genes were found across all except clade VII (*B. cytotoxicus*). For the 28 isolates screened by PCR, PCR results were consistent with identification of *hblACD* genes by BLAST (Table 3). While one study has reported presence of *hblACD* genes in all 70 tested isolates from ready-to-eat vegetables in South Korea [36], another study has found one or more *hbl* genes in 23.5 – 70.6 % of *B. cereus* group isolates from food products in Brazil [44]. The *hblA* has been detected in all 57 *B. cereus* isolates from raw vegetable samples collected in Mexico City [45]. While *hblA* and *hblD* have been detected in 72 % *B. cereus* isolates (*n* = 88) from retail spices in USA, *hblC* has been detected in 71 % of these isolates [28]. The high prevalence of the genes in the *hblACD* operon (up to 100 %) has also been reported in rice *B. cereus* isolates obtained in South Korea [37]. On the other hand, the *hblACD* genes have been found less frequently (34.5 % of 87) in *B. cereus* group isolates from fermented Korean soybean products [46]. While at least one *hbl* gene has been detected in 58.7 % of 63 *B. cereus* isolates from milk and dairy products in Brazil, all three *hbl* genes have been detected in 36.5 % of these isolates [47]. The fact that genes encoding all three toxin components are not always detected in a given isolate indicates that these genes either do not always co-exist or that false negative PCR reactions occur, e.g., due to the mismatches in primer annealing regions. Similar to our study, the *hblACD* genes have been detected in all tested *B. thuringiensis* isolates from rice in Korea [37]. On the other hand, the *hblA*C*D* genes have been detected in only 67 % of the *B. thuringiensis* isolates from retail spices in the USA [28]. These results, however, are not always directly comparable due to the differences in PCR methods used across the studies. Overall, our data support that *hblACD* genes are found across *B. cereus* group species and phylogenetic clades.

(ii) Genes encoding nonhemolytic enterotoxin (*nheABC*)

The *nheABC* genes encoding the nonhemolytic enterotoxin were identified by BLAST for all isolates (Table 2). The identification of *nheABC* genes by PCR was in agreement with BLAST results for all but 2 cases (FSL H8-0488, BGSC 4AJ1; Table 3). In these two cases BLAST search identified a significant hit, but *nheB* PCR produced a negative result. The *nheB* in these two isolates showed 1 nt mismatch with the *nheB* forward primer annealing region, however this mismatch was found also in two other isolates with positive *nheB* PCR reaction, suggesting that the sequence divergence in this region was unlikely to be the cause of false negative PCR results. Previously reported prevalence of *nheABC* genes among *B. cereus* group isolates has been generally higher (above 97 %) compared to the prevalence of *hblACD* genes in most studies, regardless of the strain source [28, 36, 44, 46]. The exception is the Korean study that reported lower prevalence of *nheABC* genes among *B. cereus* isolated from white rice (83.8 - 86.5 % of 37) [24].

(iii) Genes encoding cytotoxin K (*cytK-1* and *cytK-2*)

The gene encoding cytotoxin K (*cytK*) was detected by BLAST in approximately half of the isolates from clades III-a and III-c, and the majority of isolates from clades III-b, II, IV and VII (Table 2). The variant *cytK-1*, which is specific for *B. cytotoxicus*, was detected only in the *B. cytotoxicus* reference genome, while *cytK-2* was also detected in other genomes. Results obtained by BLAST were consistent with results obtained by PCR for 26 out of 28 tested isolates. In two cases (FSL H7-0926 and FSL K6-0267) where discrepancies were observed (Table 3), the PCR produced a positive result, while BLAST did not detect any significant hits in WGS (Additional file 3: Table S3). This may be due to the inability to detect the gene located in the region of the genome that these two draft assemblies did not cover. Fewer studies have previously evaluated the presence of *cytK*; these studies reported *cytK* in 64.7 % of 17 dairy isolates and 76.8 % of 155 *B. cereus* food isolates from Brazil [44], 70.4 % of 125 *B. cereus* isolated from milk and cereal products in Thailand [48] and 57.5 % of 87 *B. cereus* group isolates from Korean fermented soybean products [46].

### Gene encoding putative cell wall peptidase (*entFM*)

The gene encoding a putative cell wall peptidase (*entFM*) was identified by BLAST in all isolates except *B. cereus* FT9. The *entFM* was also detected in all 28 isolates tested by PCR (Table 3). The product of this gene is a putative virulence factor, reported to be involved in bacterial shape, motility, adhesion to epithelial cells, biofilm formation, and vacuolization of macrophages [49].

### Virulence gene profiles among dairy-associated isolates

The 22 dairy-associated isolates did not carry anthrax-associated virulence genes, nor genes encoding emetic toxin biosynthetic pathway. Thirteen out of 22 dairy-associated strains screened by PCR carried all eight tested diarrheal toxin genes as well as *entFM* (Table 3). The same toxin profile was most commonly identified among *B. cereus* group strains isolated from pasteurized milk and cereal products in Thailand [48]. Importantly, *hblACD*, *nheABC, cytK* and *entFM* were also found in two dairy-associated isolates, FSL K6-0267 and FSL H7-0926, which clustered in *B. weihenstephanensis* clade VI. The *hblACD*, *nheABC* and *entFM* genes were present in two reference *B. weihenstephanensis* isolates as well, initially suggesting that this psychrotolerant species may present not only a food spoilage risk, but that some strains of this species may also pose a public health concern. Subsequent experiments described in detail below, however found that an antibody-based assay did not detect hemolysin BL in these two *B. weihenstephanensis* isolates, indicating that further characterization of these isolates is needed to fully assess their pathogenicity.

### Anthrax toxin genes, diarrheal toxin genes and crystal protein-encoding genes are not exclusively associated with *B. anthracis, B. cereus*, and *B. thuringiensis* species, respectively

**Fig. 3:**
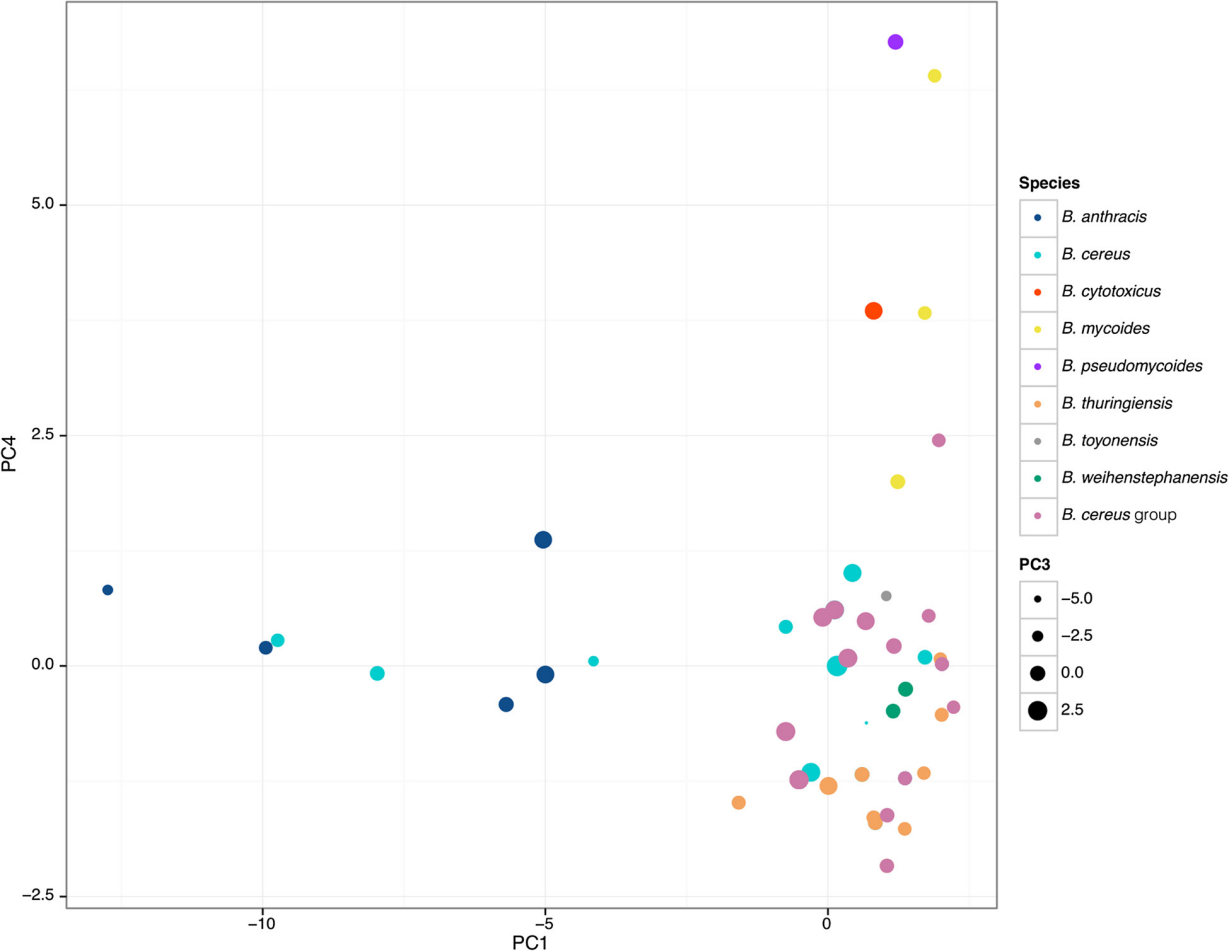
PCA clustering of *B. cereus* group isolates based on virulence gene presence/absence. PCA analysis was carried out using the data on presence/absence of 30 virulence genes that had variable presence across the analyzed 69 isolates. Reference isolates are color-coded according to the previously identified species, and all 22 dairy-associated isolates are labeled as “*B. cereus* group”. The figure demonstrates clustering of analyzed isolates based on the PC 1 (x axis), PC 4 (y axis) and PC 3 (dot size). PC 2 was omitted, as it specifically characterized a single isolate carrying genes that encode the cereulide biosynthetic pathway. The *B. cereus* and *B. anthracis* and *B. cereus* isolates carrying anthrax toxin and poly-γ-D-glutamate capsule genes form a clearly separated group on the bottom left side of the figure, while environmental isolates and *B. cytotoxicus* tend to cluster on the upper right side. Isolates carrying genes associated with diarrheal foodborne disease cluster on the bottom right side

To test for potential association of different *B. cereus* group species with certain toxin gene profiles, we analyzed the virulence gene presence/absence data by principal component analysis (PCA). The first 5 and 10 principal components (PCs) cumulatively described 69.9 % and 91.0 % of variance in the data, respectively. Clustering of isolates based on virulence gene presence/absence was visualized by plotting eigenvectors associated with PC 1 (x axis), PC 4 (y axis) and PC 3 (size of the data points) (Fig. 3). PC 2 was omitted, as it specifically characterized a single isolate carrying cereulide synthetase gene cluster (*B. cereus* AH187).

*B. cereus* group isolates formed three PCA clusters (Fig. 3) that each included isolates of multiple species. The first cluster is comprised of *B. anthracis* and *B. cereus* isolates carrying anthrax and/or capsular biosynthesis genes (negative PC1, close to neutral PC 4), the second one is comprised of nonpathogenic *B. mycoides* and *B. pseudomycoides*, as well as *B. cytotoxicus* (positive PC1 and PC4), while the majority of *B. cereus*, *B. thuringiensis* and *B. weihenstephanensis* remained unresolved in the third cluster associated with diarrheal toxin genes (close to neutral PC1 and PC4).

The first plotted principal component (PC 1) was associated with the presence of genes coding for diarrheal toxins hemolysin BL (*hblACD*), cereolysin A and B (*cerAB*), cytotoxin (*cytK*), crystal protein (*cry*), phosphatidylcholine-specific phospholipase C (*plcB*) and enterotoxic protein (*bceT*). Additionally, PC 1 was associated with absence of genes encoding anthrax toxin (*cya, lef, pagA*), poly-γ-D-glutamate capsule biosynthesis genes (*capABCDE*), global transcription regulator (*atxA*), hyaluronic acid synthase (*hasA*), hemolysin II (*hlyII*) and hemolysin II regulator (*hlyIIR*). The second plotted principal component (PC 4) was associated with presence of enterotoxic protein gene (*bceT*), and absence of genes encoding (*clo*), cytotoxin K (*cytK*), hemolysin II (*hlyII*) and hemolysin II regulator (*hlyIIR*), enterotoxic protein (*bceT*), phosphatidylinositol-specific phospholipase C (PI-PLC; *plcA*) and crystal proteins (*cry*).

Due to the inability to resolve the *B. cereus*, *B. thuringiensis* and *B. weihenstephanensis* based on the presence/absence of virulence genes, we further investigated the production of hemolysin BL and nonhemolytic enterotoxin and their association with WGS phylogenetic clades and sequence polymorphism on the 28 strains that were physically available for phenotypic tests. Details of this analysis are discussed in the following sections.

### Presence of *hblACD* and *nheABC* is not sufficient for production of detectable hemolysin BL and nonhemolytic enterotoxin Nhe

To assess the congruence of *hblACD* and *nheABC* presence with toxin production, and to identify gene polymorphisms responsible for potential inconsistencies, we examined the 22 dairy-associated *B. cereus* group isolates sequenced in this study, five *B. thuringiensis* reference isolates and *B. cereus* ATCC 14579, for detectable levels of hemolysin BL and nonhemolytic enterotoxin Nhe using Duopath Cereus Enterotoxins kit (Table 3). All tested isolates carried the *nheABC* genes, and all of them also produced the Nhe toxin at detectable levels (Table 3). In contrast, just 18 out of 28 isolates carried *hblACD* genes. Hemolysin BL was detected in only 13 of the 18 isolates that had the *hblACD* genes present, and in none of the isolates that did not have *hblACD*. Future studies assessing transcription of *hblACD* and using other phenotypic methods (e.g., tissues culture assays; ELISA assays with other [e.g., polyclonal antibodies]) will be needed to explain why some isolates that carry the *hblACD* do not express the HBL toxin.

Previous studies have confirmed the presence of *nheABC* in 83.8-86.5 % *B. cereus* and *B. thuringiensis* isolates from Korean white rice samples, but only 64.9 % of them produced NheA [37]. The *hblACD* genes were present in 83.8-94.6 % of these white rice isolates; yet only 78.4 % of them produced HblC [37]. Reis et al. [47] have reported 23 out of 63 isolated strains carrying all three *hbl* genes, but only 20 of these producing the hemolysin BL toxin. Data from these studies, as well as the present study suggest that the presence of *hblACD* genes may not be sufficient for production of detectable hemolysin BL. The hemolysin BL toxin may not be detected because i) the *hblC* is not transcribed, ii) transcribed mRNA is not translated, iii) protein is not exported, or iv) the protein was not in the configuration detectable by monoclonal antibody from Duopath Cereus Enterotoxins kit. These results demonstrate that presence of *hblACD* genes alone does not predict the production of hemolysin BL, suggesting that it may not be possible to accurately predict the risk for a *B. cereus* group isolate to cause a diarrheal disease based on the gene presence alone.

### Isolates with a truncated *hblA* variant or specific amino acid substitutions in *hblC* or *hblD* genes do not express detectable hemolysin BL

In order to explain the discrepancies between *hblABC* gene presence and hemolysin BL production in five isolates (FSL H7-0926, FSL H8-0482, FSL K6-0267, FSL M8-0117 and *B. thuringiensis* BGSC 4Y1), we have examined the *hbl* genes and individual gene amino acid sequence variability. The 466 aa long *hblB* gene (characteristic for *hbl-I* operon [43]) was not identified in two dairy-associated *B. weihenstephanensis* isolates (assigned to this species based on WGS phylogeny) that carried *hblACD* genes, but did not produce detectable hemolysin BL. Absence of *hblB* and presence of *hblACD* was also observed in *B. weihenstephanensis* KBAB4, *B. cereus* 17256 W and *B. mycoides* 219298. Another *B. weihenstephanensis* isolate (WSBC 10204) carried all four *hbl* genes, including *hblA* and *hblB*. The *hblB* is located after the stem loop structure in *hbl* operon, which may regulate its co-transcription with the rest of the genes in the *hbl* operon [43]. The role of *hblB* is not well understood yet, however, a mutant lacking *hbl-I* operon has previously been demonstrated to be less cytotoxic compared to a wild type strain carrying both operons [43]. The three *B. weihenstephanensis* isolates that lacked *hblB* also had two unique 2-aa insertions (on positions 34-35 and 246-247) and a 2-aa deletion (on position 234-235) in *hblA*. The fourth *B. weihenstephanensis* isolate (WSBC 10204), however, did not have these insertions or deletion in *hblA*. Overall, these observations provide a possible mechanistic explanation (i.e., absence of *hblB* and/or unique mutations in *hblA*) for why some *B. weihenstephanensis* may not show production of hemolysin BL detectable by the Duopath kit. Further studies, including creation of appropriate mutant strains, will be needed to provide more definitive insights.

Specific *hblACD* mutations resulting in amino acid substitutions were observed in the dairy isolate FSL M8-0117 and in *B. thuringiensis* BGSC 4Y1, which both carry the *hblACD* genes, but did not produce detectable hemolysin BL. For isolate FSL M8-0117 the threonine at the 410^th^ position of *hblC* (lytic component L2) was substituted with alanine. The polar threonine is commonly found in catalytic sites; therefore its substitution with hydrophobic alanine may impact protein functionality [50]. Similarly, a threonine at the 358^th^ position of *B. thuringiensis* BGSC 4Y1 *hblC* gene was replaced with an electrically charged lysine. A non-neutral substitution was also identified in *hblD* (lytic component L1) of *B. thuringiensis* BGSC 4Y1, where a histidine at the 27^th^ position was replaced with glutamine. The electrically charged histidine does not substitute well with any other amino acid and is often part of binding or active sites [50]. Its replacement with polar glutamine may therefore affect the toxin assembly and activity. Neither of these substitutions was observed in isolates producing detectable hemolysin BL toxin. Single amino acid substitutions have been previously shown to abolish monoclonal antibody reactivity with toxin components in other organisms, such as *B. anthracis* [51]. Substitutions in specific asparagine and proline residues of anthrax protective antigen have been demonstrated to reduce the cytotoxicity and antibody binding, while specific lysine, leucine and tyrosine residues have affected only the antibody binding [51]. It is not yet understood whether observed mutations abolish fully functioning toxin expression, or just its detection by the monoclonal antibodies used in Duopath kit [52]. Future studies are needed to address these questions in order to allow for sequence-based prediction of the toxicity associated with different *hbl* gene variants.

### WGS clade IV is associated with hemolysin BL toxin production

Isolates that were tested for toxin production were distributed among clades II to VI. All tested isolates from clades III-b, III-c and VI did not produce a detectable hemolysin BL toxin. Clades II, III-a and V all included isolates with and without production of hemolysin BL; 2 of 6, 1 of 2, and 1 of 2 tested isolates, respectively, produced hemolysin BL. All tested isolates from phylogenetic clade IV tested positive for hemolysin BL production. Hemolysin BL production was significantly (*p* = 0.0001) associated with this clade, which is consistent with a previous report [34].

## Conclusions

WGS-based phylogenetic classification of *B. cereus* group isolates showed better association with presence of toxin genes and toxin production than traditional taxonomic species classification. Clade classification along with the identification of virulence gene presence/absence and allelic variation in hemolysin BL-encoding genes thus appears to provide for better characterization of isolates with regard to their likelihood of causing diarrheal foodborne and other diseases. Overall, our findings support previous studies (e.g. [33, 34]) that have emphasized the need to re-evaluate the taxonomy of the *B. cereus* group, specifically of the species *B. cereus* and *B. thuringiensis*, which both were found in multiple clades. In the meantime, our data support a previous proposal that *B. cereus* group isolate nomenclature should include species and phylogenetic clade. The clade classification can be achieved by WGS as well as *rpoB*, *panC* or MLST sequencing, albeit at a lower level of phylogenetic resolution compared to WGS.

## Methods

### Bacterial isolates

Twenty-two *B. cereus* group isolates from dairy-associated sources were selected for WGS, from an existing Cornell Food Safety Laboratory strain collection that includes 3608 *Bacillus* isolates (as of 28 March 2016) that have been classified, as previously described [53, 54], by sequencing of a 632 nt internal fragment of *rpoB*, which encodes the β-subunit of RNA polymerase. Detailed data for all isolates are available in the Food Microbe Tracker (FMT) database [54]. Overall, isolates were selected to represent different combinations of *rpoB* AT, toxin gene profiles, and hemolytic potential (Table 3), based on initial screen of 114 isolates that had been classified as *B. cereus* group based on *rpoB* sequence data [55] (Table 3). Two selected isolates (FSL W8-0520 and FSL W8-0523, both *rpoB* AT481) shared the same profiles. Isolate sources included raw milk, dairy processing facility environments, and other dairy sources (Table 3).

An additional 47 reference genomes of *B. cereus* group isolates from human clinical cases, environment, and food or plant origin were extracted from NCBI Genome database and included in the analysis to represent the phylogenetic diversity of *B. cereus* group species (Additional file 1: Table S1).

### *rpoB* allelic typing, virulence gene PCR screening and hemolysis

The molecular typing of isolates in the FMT database had been performed by PCR amplification and sequencing of a 632 nt long internal fragment of *rpoB*, which has been used for *rpoB* allelic (AT) assignment, as described previously [53, 55]. Novel *rpoB* ATs were assigned to every new variant of the 632 nt *rpoB* gene fragment that differed from any *rpoB* sequence in the existing database by at least 1 nt. The 632 nt *rpoB* sequences of the 22 FSL isolates were used for construction of a maximum likelihood phylogenetic tree in RaxML version 8 using general time-reversible (GTR) model with gamma distributed and invariant sites (GAMMAI), and 1000 bootstrap repetitions [56]. The phylogenetic tree was edited in FigTree v.1.4.2, and deposited on Figshare (https://figshare.com/s/7f5965d498bd33ccaac4).

Isolates were further characterized by PCR screening for the presence of genes encoding hemolysin BL (*hblA, hblC, hblD*), the nonhemolytic enterotoxin (*nheA, nheB, nheC*), and cytotoxin K (*cytK*) and FM toxin (*entFM*) [57, 58].

The ability of isolates to induce hemolysis was evaluated by plating overnight cultures onto Trypticase soy agar (TSA) supplemented with 5 % sheep blood (BBL, New Jersey, USA), followed by incubation at 35 °C for 24 h according to the FDA’s Bacteriological Analytical Manual. Alpha hemolysis was determined based on the dark green agar discoloration around and under the colonies, and beta hemolysis based on the transparent zones around and under the colonies (Table 3).

### Detection of hemolysin BL and nonhemolytic toxin production

Hemolysin BL and nonhemolytic toxin (Nhe) production was detected using Duopath Cereus Enterotoxins immunological lateral flow assay (Merck) for all 22 FSL *B. cereus* group isolates, *B. cereus* ATCC 14579, and five *B. thuringiensis* isolates (BGSC 4Y1, BGSC 4CC1, BGSC 4BD1, BGSC 4BA1). Duopath test kits were used as specified by manufacturer’s instructions, to detect toxin production in bacterial cultures grown in Brain-heart infusion (BHI) broth at 37 °C for 20-24 h. The Duopath Cereus Enterotoxins kit is able to detect the NheB component of the Nhe enterotoxin at concentration of ≥6 ng/ml and Hbl-L(2) component of hemolysin BL at concentrations ≥20 ng/ml [52]. Results reported represent two biological replicates. Associations of hemolysin BL production with WGS phylogenetic clades were tested using Fisher’s exact test in R version 3.2.2 [59].

### DNA extraction, library preparation and Illumina sequencing

Frozen cultures (-80 °C) stored in 15 % v/v glycerol-BHI media were streaked on BHI agar and incubated for 24 h at 32 °C. The DNA of isolates was extracted using QIAamp DNA MiniKit (Qiagen, Valencia, CA) with a 30 min Gram positive pre-treatment in 80 μl of 20 mg/ml lysozyme at 56 °C, and longer centrifugation times at lower centrifugal force as previously reported by our group [60]. DNA was eluted in 50 μl Tris-HCl (pH 8.0), and purity was assessed spectrophotometrically using a Nanodrop. Double-stranded DNA (dsDNA) was quantified with Picogreen (Invitrogen, Palsley, UK), normalized to a concentration of 0.2 ng/μl, and used to prepare a sequencing library with Nextera XT DNA Sample Preparation kit and Nextera XT Index Kit with 96 indices (Illumina, Inc. San Diego, CA). Subsequently, samples were pooled and sequenced in 2 lanes of a HiSeq 2500 rapid run with 2 x 100 bp paired-end sequencing with estimated 67x coverage (Genomics Facility of the Cornell University Institute of Biotechnology).

### Genome assembly

Trimmomatic v0.32 [61] was used to trim Illumina adapters, as well as low quality bases and reads, according to the program’s quick start parameters for paired-end reads. Short read quality was evaluated using FastQC v0.11.2 [62]. Processed sequence reads were assembled with SPAdes v3.0.0 with the suggested k-mer set for bacterial genome assembly and the best assembly was used in the analysis [63]. Quality of assemblies was verified by QUAST [64] and the average genome coverage was determined using SAMtools [65].

### Multilocus sequence typing (MLST)

MLST sequence types were determined for 22 FLS isolates by running SRST2 v0.1.6 [66] on short read fastq.gz files, using the *B. cereus* allele and sequence type definition database obtained from the PubMLST database [35] using a getmlst.py script. Unidentified allelic types and sequence types were extracted from the whole genome sequences using BLAST and submitted to the PubMLST database [35]. The concatenated sequences of 7 MLST loci for the 22 FSL isolates were used for construction of a maximum likelihood phylogenetic tree in RaxML version 8 using general time-reversible (GTR) model with gamma distributed and invariant sites (GAMMAI), and 1000 bootstrap repetitions [56]. The phylogenetic tree was edited in FigTree v.1.4.2, and deposited on Figshare (https://figshare.com/s/7f5965d498bd33ccaac4).

### Phylogenetic classification based on *panC* sequence

The *panC* gene encoding pantoate-beta-alanine ligase was extracted from analyzed set of WGS by BLAST using *B. cereus* ATCC 14579 pantoate-beta-alanine ligase gene (NCBI Gene ID: 1203890) as a reference sequence. Extracted *panC* sequences were used for classification of isolates into seven phylogenetic groups proposed by Guinebretière et al. [33, 34] using their online classification tool [67]. The identified phylogenetic groups, I to VII, were associated with WGS clades identified in our study (Fig. 1).

### Single nucleotide polymorphism (SNP) calling and phylogenetic analysis

The 22 draft genomes sequenced in this study and 47 closed genomes extracted from NCBI Genome were used to build a SNP-based phylogeny with kSNP v2 using kmer size 21, as determined by Kchooser [68]. The core SNPs were aligned in MEGA v6.06 [69] with MUSCLE algorithm and used to build the maximum likelihood phylogeny in RaxML under general time-reversible model with gamma distributed sites (GTRGAMMA) and 1000 bootstrap repetitions. The phylogenetic tree was edited in FigTree v.1.4.2, and deposited on Figshare (https://figshare.com/s/7f5965d498bd33ccaac4). The absolute core SNP distance matrix was calculated using PAUP 4.0a147 (Additional file 4: Table S4).

### Virulence gene presence/absence and PCA analysis

Thirty-five reference virulence protein sequences (Additional file 2: Table S2) were queried against the whole genome sequence database (*n* = 69) assembled here, using tblastn function of standalone BLAST to investigate the presence/absence of virulence gene homologues. The R script was used to filter BLAST hits with >70 % alignment length, >50 % amino acid sequence identity and <10^-5^ e-value. The 50 % amino acid identity cut-off was determined based on the congruence of the BLAST search with results of the PCR screening (Fig. 2). Due to the high degree of evolutionary divergence of crystal protein (*cry*) genes of *B. thuringiensis,* these were identified using an Endotoxin_M hidden Markov model (HMM) extracted from Pfam database (PF00555), a custom-built hidden Markov model (HMM) using 10 *cry* variants representing the different 1° domains available in the *B. thuringiensis* nomenclature database [41], and RAST [42]. To build a custom HMM, amino acid sequences were aligned in MEGA v6.06 using MUSCLE algorithm, and were used to run hmmbuild in HMMER v3.1b2 [70]. The assembled chromosomal and plasmid sequences were joined as pseudochromosomes using a shell script, and translated in all 6 reading frames by running transeq in EMBOSS v6.5.7 [71]. Translated genomes were used as a subject database in hmmsearch (HMMER v3.1b2). To ensure the specificity, the conservative threshold e-value and HMM score were determined based on the output of the HMM search against the aligned *cry* variants that were used for building the model (Additional file 5: HMM_cry_log.sh).

The binary matrix capturing the presence/absence of 36 virulence genes was reduced to 30 genes with variable presence/absence data, and this reduced matrix was used in PCA analysis (R package “stats”; routine “prcomp”). The principal components were plotted using R package “ggplot2”.

### NCBI accession numbers

Trimmed WGS reads and assembled genomes were submitted to the SRA and GenBank, under the BioProject ID PRJNA288461. Accession numbers are listed in Table 1.

## Additional files

Additional file 1: Table S1. List of publicly available closed genomes included in this study. (XLSX 43 kb) Additional file 2: Table S2. Virulence-associated genes included in the comparative genomic analysis of Bacillus cereus group isolates. (XLSX 33 kb) Additional file 3: Table S3. Presence/absence of 36 virulence genes in whole genome sequences of 69 B. cereus group isolates. (XLSX 19 kb) Additional file 4: Table S4. Absolute core genome SNP distance for 69 B. cereus group isolates. (XLSX 61 kb) Additional file 5. HMM_cry_log.sh. (SH 932 bytes)
